# Using Intradermal Rabies Vaccine to Boost Immunity in People with Low Rabies Antibody Levels

**DOI:** 10.4061/2011/601789

**Published:** 2011-05-25

**Authors:** David Brown, Anthony R. Fooks, Martin Schweiger

**Affiliations:** ^1^Virus Reference Department, Centre for Infections, HPA, London NW9 5EQ, UK; ^2^Animal Health and Veterinary Laboratories Agency (AHVLA), Weybridge, Woodham Lane, Addlestone, Surrey KT15 3NB, UK; ^3^Wildlife Zoonoses and Vector-Borne Diseases Research Group, University of Liverpool, Liverpool, UK; ^4^West Yorkshire Health Protection Unit, HPA, Leeds LS15 7TR, UK

## Abstract

Intradermal rabies vaccine is recommended by the World Health Organisation, but not all countries, including England, follow this recommendation. A group of 12 adults in England previously given pre-exposure intradermal rabies vaccine were considered to be non-immune to rabies because their rabies antibody titres were known to be less than 0.5 IU/mL. A cohort study examined the immunizing effect of increasing the participants' cumulative dose of intradermal rabies to 2.0 IU. All patients subsequently demonstrated rabies antibody levels >0.5 IU·mL supporting evidence of adequate sero-conversion. No adverse effects of intradermal rabies vaccine boosting were noted. Within the limits of a small study the findings support the hypothesis that adequate levels of rabies antibody can be achieved by a schedule of intradermal injections delivered on at least three occasions with a cumulative rabies vaccine dose of 2.0 IU.

## 1. Introduction

A study undertaken in 2007 on the duration of the immunogenicity of intradermal rabies vaccine demonstrated titres of rabies antibodies consistent with a protective response a decade or more after immunization with human diploid cell vaccine [1]. Twenty one of the 89 participants in that study failed to demonstrate titres of rabies antibodies greater than 0.5 IU/mL and were considered not to be adequately protected against rabies. Of the twenty one, none had received a cumulative dose of intradermal rabies vaccine greater than 1 IU nor had they received rabies intradermal vaccination on more than two occasions. Based on this observation, we hypothesised that the production of levels of rabies antibodies that can be correlated with protective efficacy requires a minimum cumulative dose of 2.0 IU of rabies vaccine administered intradermally over not less than three separate occasions. An antibody titre of greater than or equal to 0.5 IU/mL was considered indicative of seroconversion, providing an adequate titre, in line with the World Health Organization (WHO) recommendations [2].

The study reported in this paper was undertaken in order to examine that hypothesis.

## 2. Method

This study was based on inviting the 21 nonresponding participants of the first study to provide 4 mL blood samples to confirm the previously determined antibody titres. Twelve of the 21 were both willing and able to participate. They were given booster doses by the intradermal route to bring them up to a life time cumulative dose of 2.0 IU of rabies vaccine. In all cases this proved to require 1.0 IU of rabies vaccine. After an interval of approximately 6 weeks a second 4 mL blood sample was obtained. Antibody levels were measured from “blinded” blood samples to reduce the risk of bias. All serum samples were tested by the fluorescent antibody virus neutralisation test [3]. This test is regarded in the UK as the gold standard, with a high sensitivity for detecting postimmunisation antibody levels.

Prior to the study commencing approval was sought from the Health Protection Agency's Research Sponsorship Review Group, the West Yorkshire Primary Care Research and Development Unit for Research Governance Approval, the National Research Ethics Service for ethical approval and the Medicines and Healthcare products Regulatory Agency for a clinical trial authorization. This approval process commenced in August 2008 and concluded in March 2009. The Eudract Number granted was 2008-005465-56.

Following the protocol developed for this study all 21 possible participants were sent a postal invitation to participate in this study. This invitation informed them of the purpose of the study and what was expected of them. A form was included on which the possible participant was asked to indicate if they wished to discuss taking part and asking them to provide their preferred contact details. This form could be returned in an accompanying stamped addressed envelope. Returned forms were followed up with a telephone call made by one of the authors to confirm that the participant was still eligible and willing to participate. Arrangements to collect the blood samples and to give the booster dose of rabies vaccine were agreed by telephone and followed up in writing. In most cases the work was undertaken at the Leeds Overseas Travellers Clinic, Yorkshire, UK, where the participant had received their original course of pre-exposure rabies vaccine prior to travel. 

At the first of the two clinic visits the participant was again advised about the study and asked to sign a consent form. A 4 mL venous blood sample was taken and collected in a plain glass tube and stored at 4°C. Two separate 0.5 IU doses of rabies vaccine were then administered by the intradermal route over the left deltoid muscle, the injections being between 2 and 3 cms apart. The vaccine used was Verorab (Pasteur Merieux) Lot number B0529, due to expire in May 2010. This vaccine is recommended for intradermal use by WHO [4]. Verorab is prepared using a cell culture technique using cells originally harvested from African Green Monkeys. All blood was collected by one of the authors who also gave all the intradermal immunizations to ensure consistency of technique. After an interval of six to eight weeks participants were seen again. Enquiries were made about any possible side effects from the immunization, and a second 4 mL venous blood sample was collected. Antibody results were matched against the list of participants to allow tabulation and review.

## 3. Subjects and Recruitment

The only people initially eligible to participate were the 21 participants from the previous study who had demonstrated antibody titres less than 0.5 IU/mL. Five people did not reply to the letter of invitation and could not be traced. Two had contraindications to participation in the form of additional intramuscular doses of rabies vaccine to protect them during travel to rabies risk areas during the two years since the first study. One had developed a serious illness and was now taking long-term steroids. Only one previous participant expressly refused to participate in this study.

Twelve previous participants agreed to take part again and on questioning did not appear to have any contra-indication from doing so. No financial inducement was paid to the participants. All participants were, however, assured that if any problem did arise as a result of their participation there was a helpline telephone number to call. They were also informed in writing that the researchers were indemnified should there be any serious untoward consequences from participation. The eligibility criteria used were participation in the previous study, having a documented antibody titre of less than 0.5 IU/mL, not having received any rabies vaccine by any route since the first study, and not having any condition that might impair immunity.

## 4. Outcome Measures

The main outcome measure sought was the rabies antibody titre following the booster dose of rabies vaccine. A secondary outcome measure was the degree of change in antibody titre since the previous study. Participants were also all asked if they experienced any untoward effects of the booster dose.

## 5. Statistical Analysis

Laboratory results were tabulated. The key determination was the proportion of participants who, following booster intradermal rabies vaccine demonstrated antibody titres equal to or greater than 0.5 IU/mL. The mean rise in antibody titre was also determined and examined for the effects of age or gender.

## 6. Results and Discussion

The key results are displayed in Table 1.

Of the 12 participants the majority (75%) were women. Ages ranged from 20 to 71 years of age. All had received a total of 0.4 mL of previously available vaccines which is equivalent to 1 IU of rabies vaccine. Six of the 12 participants had received their previous rabies vaccine at a single clinic visit, five on two clinic visits, and one on four clinic visits. As shown in Table 1 pre-booster antibody titres are consistent with the previous estimation two years earlier. All participants demonstrated postbooster antibody titres higher than the minimum considered consistent with immunity to rabies. The mean pre-booster titre was 0.18 (CI 0.12–0.25), and the mean postbooster titre was 17.33 (CI1.48–33.19). The mean antibody rise was 17.15 IU/mL. The range of antibody rises was very wide, varying from 1.75 to 69.93 IU/mL. The 95% confidence intervals are 1.326–32.973 IU/mL. 

**Figure 1:**
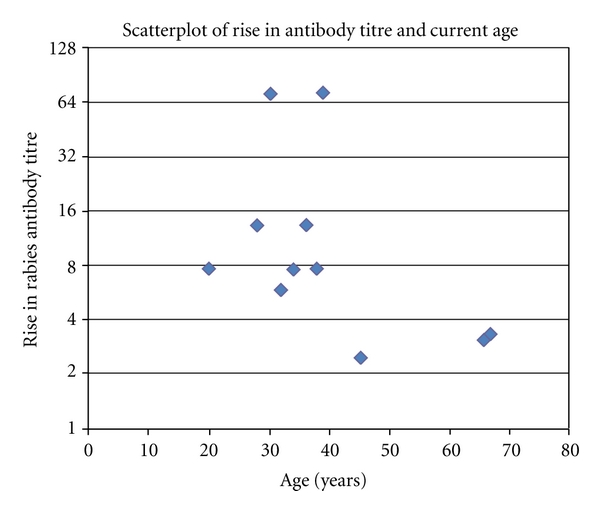
Scatter plot of antibody titre rise and age in years.

The two results with increases in antibody titre of almost 70 skew the results and lie outside the 95% confidence intervals. Using a log transformation of the difference between pre- and posttitre levels gives a highly significant *P* value of <.01; the post-booster titre levels were significantly larger than the pre-booster titre levels (see Figure 1).

The most notable increases in antibody titre were observed in two women in their 30 s who had received their last rabies immunization between 4 and 5 years previously. Apart from age and gender no explanation for their high antibody response was determined. If these two results are excluded, the results did not show any obvious gender difference.

**Figure 2:**
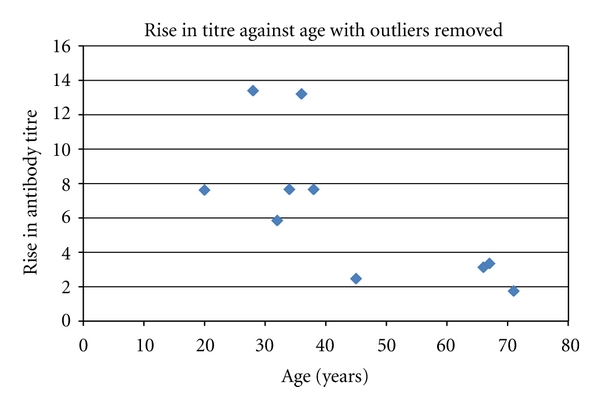
Scatter plot of rise in tire after boosting with outliers removed against age.

The apparent decline in antibody response with age is shown even if the two outliers are removed from the analysis as shown in Figure 2.

**Figure 3:**
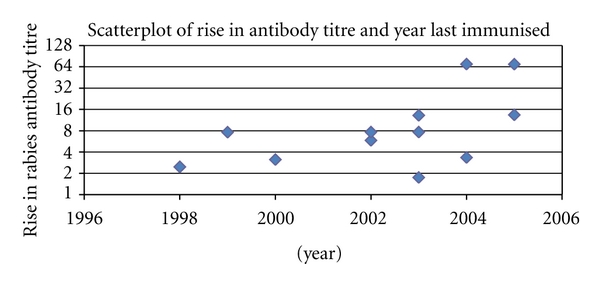
Scatter plot of rise in antibody titre and year last immunized.

**Figure 4:**
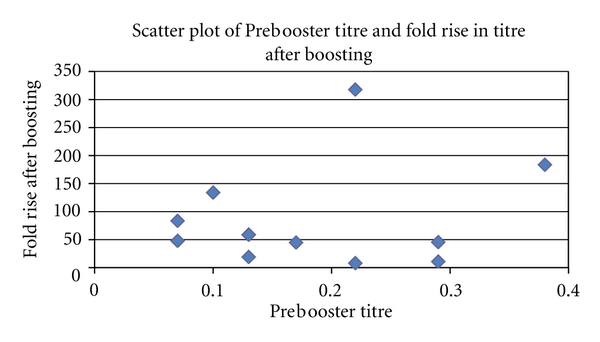
Scatter plot of pre-booster tire and fold rise in titre after boosting.

Figure 3 suggests that there was a decline in antibody response after 10 years. The titre immediately prior to boosting did not appear to have any direct effect on the titre achieved after boosting immunization. This can be observed in Figure 4.

Participant 2 disclosed after the second blood sample that she had received chemotherapy for breast cancer since the previous study, if this had been disclosed earlier, she would have been excluded from the study. Interestingly, she demonstrated a good antibody response to the intradermal immunization. None of the participants reported any noticeable side effects from the immunization. None of the participants called the telephone helpline offered at entry to the study. Rabies remains a serious global challenge with an estimated 55,000 deaths each year [5]. The availability of an effective vaccine is restricted by total vaccine production and relatively high cost. If intradermal rabies immunization schedules were introduced for pre-exposure prophylaxis, it would both increase the global supply of vaccine doses and reduce the cost per person immunised.

It is not possible to accurately determine the level of rabies virus neutralizing antibody adequate to provide protective immunity for humans. The World Health Organization regards 0.5 IU/mL as an adequate level of antibody after vaccination [2] and that has been accepted by the authors of this paper, providing the basis for our confidence that 12 previously inadequately protected individuals now have adequate protective antibody levels. Previous work undertaken by the authors suggests that this protection should last for at least ten years [1].

Versions of rabies vaccine previously used on the patients in this study contained 2.5 IU/mL. Many previous reports that discuss the effectiveness of both intramuscular and intradermal vaccines quote the dose administered in volume terms. The vaccine available for this study, Verorab, has twice the concentration containing 5 IU/mL, which is supplied in 0.5 mL vials each containing 2.5 IU of rabies vaccine. In order to avoid confusion this paper has quoted the dose in International Units rather than volume administered.

There were only 12 participants in this study so there is a possibility that they do not represent the true population of responders to boosting with intradermal vaccine. Calculation of the 95% confidence intervals of a small sample with a skewed distribution results, as in this case, in wide confidence intervals. Participants ranged widely in age and in time since previous rabies immunization. All developed an effective rabies antibody response. All 12 participants in this study demonstrated a good response to the booster doses supporting the hypothesis that antibody levels consistent with immunity to rabies are likely to be achieved if a total of 2 IU of rabies vaccine have been administered over three or more occasions. In this study the last dose had been administered within the previous 10 years. Taken with the findings of long-lasting immunity following intradermal rabies vaccine in our earlier study [1], there is evidence to support a pre-exposure intradermal rabies immunization schedule based on delivering 2.0 IU spread over three doses. The interval between these three initial doses could be based on the observations of Thai studies which demonstrated effective protection with doses given on days 0, 7, and 28 described by Strady et al. [6] who used the intramuscular route and Kamoltham et al. [7] who used purified chick embryo cell vaccine given intradermally. A study by Naraporn et al. [8] examined the immune response to rabies booster immunization after an interval of 5 years and showed that all 36 patients who completed the study at 28 days had a good anamnestic antibody response to two intradermal booster injections of purified duck embryo cell vaccine given 3 days apart. Malerczyk et al. [9] report a study in 15 German veterinarians who had received purified chick cell embryo cell vaccine 14 years previously who were boosted with intramuscular purified chick cell embryo vaccine. All ten veterinarians who submitted blood samples after immunization demonstrated a good anamnestic response. Suwansrinon et al. [10] describe a study in Thailand in which 53 patients who had received rabies immunization between 10 and 20 years previously were given two 0.9 IU doses of Vero cell rabies vaccine three days apart. Two weeks after immunization all had antibody levels that exceeded the critical threshold considered to provide adequate immunity of 0.5 IU/mL.

The use of intradermal human diploid cell rabies vaccine for boosting purposes was examined in a study undertaken in 1987 which followed up 40 laboratory workers who had been given intradermal rabies vaccine in 3 separate doses totaling 0.75 IU [11]. Twenty of these workers demonstrated titres considered to be protective at 1 year, but by 2 years 5 were considered to have unprotective levels. Intradermal boosters given to 4 of these 5 laboratory workers produced high titres of rabies antibody. That study recommended serological testing every two years with a booster dose given to those with what are regarded as unprotective titre levels.

The observations of the study being reported in this report suggest that increasing the initial course to a total of 2 IU given over three clinic visits will provide effective rabies protection. Review of the literature and our previous study suggest that adequate immunity will be maintained for at least 10 years without the need for expensive serological testing or boosting. It is reasonable to conclude that immunization with at least 2 IU of rabies vaccine by the intradermal route should result in an antibody titre that will provide protection. This regimen could preserve the limited stocks of rabies biologicals, including rabies immunoglobulin. It is suggested that the time intervals used between doses can be based on the work of Strady et al. [6], with an initial dose of 1 IU on day 0 followed by 0.5 IU on days 7 and 28. These doses are relatively easy to translate into volume terms based on the concentration of the vaccine available for use.

No adverse effects were reported after immunization supporting the hypothesis that boosters of two intradermal 0.5 IU Vero cell-derived rabies vaccine injections can safely be coadministered after 10 years with immunity maintained at an adequate level.

This study did not examine the effect of giving a single 0.5 IU dose of rabies vaccine as a 10-yearly booster; however the good level of response suggests that further cost and vaccine savings could be safely achieved. Further research to assess the increase in antibody titre following a single 0.5 IU dose of rabies vaccine is advisable. The absence of any serious side effects in this small sample of 12 patients given intradermal rabies vaccine is reassuring and helps to give confidence that the intradermal immunization route is not only effective but also safe [5].

##  Funding

A. R. Fooks was funded by the UK Department for Environment, Food and Rural Affairs (Grant SEV 3500). The rabies vaccine used was paid for by the West Yorkshire Health Protection Unit of the Health Protection Agency. The Clinical Trials Approval application fee was paid for by James Taylor & Son, (Bespoke shoemakers). M. Schweiger is a member of staff of the West Yorkshire Health Protection Unit; the other funders had no role in study design, data collection and analysis, decision to publish, or preparation of the paper.

##  Conflict of Interests

The authors have declared that no competing interests exist.

## Figures and Tables

**Table 1:** Summary of the results obtained for each participant.

Participant number	Age	Gender	Year last immunised	Vaccine administered on days	Antibody titre in 2007	Pre-booster titre, 2009	Post-booster titre, 2009	Rise in antibody titre following immunisation
1	38	F	2003	0, 54	0.38	0.13	7.79	7.66
2	66	F	2000	0, 28	0.38	0.29	3.42	3.13
3	32	F	2002	0	0.06	0.07	5.92	5.85
4	67	M	2004	0	0.29	0.07	3.42	3.35
5	71	M	2003	0, 28	0.38	0.22	1.97	1.75
6	45	F	1998	0, 28	0.22	0.13	2.60	2.47
7	36	F	2003	0	0.38	0.29	13.5	13.21
8	30	F	2004	0	0.29	0.22	70.15	69.93
9	39	F	2005	0, 28	0.38	0.38	70.15	69.77
10	34	F	2002	0, 7, 21, 28	0.38	0.13	7.79	7.66
11	20	M	1999	0	0.38	0.17	7.79	7.62
12	28	F	2005	0	0.38	0.10	13.5	13.4

All titre results expressed in IU/mL.
